# Transcriptome Analysis of Newly Emerged Honeybees Exposure to Sublethal Carbendazim During Larval Stage

**DOI:** 10.3389/fgene.2018.00426

**Published:** 2018-10-08

**Authors:** Kang Wang, Rong-Li Fan, Wen-Na Ji, Wen-Wen Zhang, Xiao-Mei Chen, Shuang Wang, Ling Yin, Fu-Chao Gao, Guo-Hong Chen, Ting Ji

**Affiliations:** ^1^College of Animal Science and Technology, Yangzhou University, Yangzhou, China; ^2^College of Horticulture and Plant Protection, Yangzhou University, Yangzhou, China; ^3^Jiangsu Agri-animal Husbandry Vocational College, Taizhou, China; ^4^Mudanjiang Branch of Heilongjiang Academy of Agricultural Sciences, Harbin, China

**Keywords:** honeybee, sublethal dose effects, carbendazim, brain development, transcriptome

## Abstract

There are increasing concerns regarding the impact of agrochemical pesticides on non-target organisms. Pesticides could cause honeybee abnormal development in response to neurotoxins such as neonicotinoid. However, knowledge of carbendazim, a widespread fungicide in beekeeping practice, influencing on honeybee (*Apis mellifera* L.) brain development is lacking. Large-scale transcriptome approaches were applied to determine the changes in global gene expression in the brains of newly emerged honeybees after carbendazim exposure during the larval stage. To further understand the effects of carbendazim on the brain development of honeybees, the functions of differentially expressed genes were compared between the treatment and control groups. We found that neuroregulatory genes were down-regulated after carbendazim exposure, which suggest the neurotoxic effects of this fungicide on honeybee nervous system. Carbendazim exposure also altered the expression of genes implicated in metabolism, transport, sensor, and hormone. Notably, larvae in the carbendazim-treated group observed longer time to shift into the dormant pupal state than the control group. Moreover, a low juvenile hormone and high ecdysone titers were found in the treatment group compared to control group. The data is the first report of neurotoxic effects on honeybee caused by carbendazim, and the sublethal carbendazim may disturb honeybee development and is a potential chemical threating the honeybee colonies.

## Introduction

Bees are pollinators of great social, economic, and ecological importance in agricultural production and biodiversity maintenance (Potts et al., 2010). Pesticide application has considerably reduced bee colonies in many countries, and this issue is perceived as a threat to biosecurity and environmental health (Goulson et al., 2015). The use of low-toxicity pesticides has alleviated the acute effects of highly toxic pesticides to some extent, but there are increasing concerns regarding the sublethal effects of low-toxicity pesticides on non-target organisms (Desneux et al., 2007; Doublet et al., 2015; Fauser et al., 2017).

Traditionally, the classical laboratory method for estimating the side effects of chemicals on beneficial arthropods was to determine a median lethal dose (LD_50_) or lethal concentration. However, the estimated lethal dose during acute toxicity tests may only be a partial measure of the deleterious effects. In addition to direct mortality induced by pesticides, their sublethal effects on arthropod physiology and behavior must be considered for a complete analysis of their impact (Desneux et al., 2007). Previous studies have shown that pesticides can affect honeybee (*Apis mellifera* L.) behavior, neurophysiology, biochemistry, longevity, development, fecundity, immunity, orientation, mobility, learning, oviposition, and other characteristics (Papaefthimiou and Theophilidis, 2001; Desneux et al., 2007; Fischer et al., 2014; Sandrock et al., 2014; Kessler et al., 2015; Sánchez-Bayo et al., 2016; Wu et al., 2017). Different types of pesticides have distinct mechanisms and target organisms. Neurotoxicant and neonicotinoids are known to severely disturb the honeybee nervous system, including synaptic transmission, navigation, and learning ability (Jin et al., 2015; Stanley et al., 2016; Wu et al., 2017). These behavioral changes usually result in colony failure and they have raised public awareness regarding honeybees’ health and welfare. A 2-year ban on the usage of the three most common types of neonicotinoid was launched in 2013. Recently, Europe strengthened the ban on bee-harming pesticides in response to the concern that the toxicity of these chemicals actually increases over time.

The present study investigated the toxic effects of the broad-spectrum fungicide carbendazim on honeybees. In China, carbendazim has been used to control fungal pathogens on camellia, rape nectar plants, and other crops. In mainland China, the detection rate of carbendazim in 282 apple samples was 81.9%, which is one of the highest (Ye et al., 2016). Moreover, Zhou measured pesticide residues of 48 pollen samples collected from eight provinces in mainland China. As a fungicide with the highest detection rate (77.1%), the maximum concentration of carbendazim reached 4,516 ng/g.(Zhou et al., 2016). A survey conducted by the China Institute of Apicultural Research also showed that the detection rate of carbendazim was the highest among all pesticides identified in bee products. In fact, carbendazim residues have been found in pollen from many countries other than China (Mullin et al., 2010; Kasiotis et al., 2014; Sanchez-Bayo and Goka, 2014; David et al., 2015; Calatayud-Vernich et al., 2018). At present, migratory beekeeping has become a major way of commercial apiculture in China. Therefore, carbendazim exposure might be inevitable, and it might be transported from one polluted farm to others as bees forage and pollinate. However, there are few studies on the effects of carbendazim on honeybee. Hence, it is necessary to investigate the sublethal effects of carbendazim on the growth and development of honeybees.

In recent years, constant news on the collapse of bee colonies stimulated our attention (Cox-Foster et al., 2007; Luo et al., 2008; Van der Zee et al., 2012). Completion of honeybee genome sequencing in 2006 revealed that honeybees have relatively few genes encoding detoxifying enzymes (Honeybee Genome et al., 2006). Because carbendazim is frequently applied as a crop fungicide, it might be considered as a possible factor contributing to the mass death of bee colonies in China. Here, high-throughput RNA-sequencing (RNA-Seq) was utilized to identify and quantify the expression levels of the genes transcribed in the heads of adult honeybees whose larvae had been exposed to carbendazim. The resulting transcriptome library may help elucidate the sublethal effects of carbendazim on honeybees.

## Materials and Methods

### Honeybee and Chemicals

Honeybees (*A. mellifera*) were obtained from the Honeybee Research Institute of Yangzhou University, Yangzhou, China. Colonies were maintained in accordance with standard practices. Each colony had one young egg-laying queen and a population of nine comb frames with pollen, pupae, larvae, and honey. Carbendazim is distributed as a powder and it is soluble in organic solvents like dimethyl sulfoxide (DMSO). Here, DMSO was used because its amount was unimportant in the final test solution (0.1 %, v/v), and it is a widely accepted co-solvent in toxicology experimental research involving honeybees (Suchail et al., 2000; Faucon et al., 2005; Yang et al., 2012; Wu et al., 2017).

The comprehensive and effective stress concentrations of carbendazim applied here were based on published data from the survey of pesticide residues in 48 pollen samples collected from eight provinces in mainland China (Zhou et al., 2016). In this report, the maximum carbendazim concentration detected was 4.516 ng mg^−1^, and, therefore the rounding value of 5 ng mg^−1^ was adopted in the present study. Generally, different stress methods and times may result in discrepant conclusions, even when experimental subjects are exposed to identical concentrations in toxicology tests. Thus, a necessary pre-experiment was conducted to demonstrate that there was no apparent difference in mortality between control and 5 ng mg^−1^ carbendazim-treated groups (χ^2^ = 5.642, adjusted *P* = 0.078 > 0.05) (**Supplementary Table S1**), even at high concentrations. Hence, 5 ng mg^−1^ was considered as a reasonable and practical sublethal concentration of carbendazim to apply to honeybees in our experiment.

### Sample Preparation and *in vitro* Artificial Larval-Rearing

The sublethal effects of carbendazim on honeybees were assessed using an artificial larva-rearing system modified from previous studies (Crailsheim et al., 2013; Wu et al., 2017). Three honeybee colonies were selected for this experiment. An excluder was used to restrict the queen to deposit eggs in a specified empty frame in each test hive for 10 h, and the same experiments were conducted in the other two test hives simultaneously. After 72 h, 1-day-old larvae were collected from combs and maintained on a diet consisting of 0.5% yeast extract, 8% fresh honey, 36.5% ultrapure water, and 55% fresh royal jelly for 1 day. This formulation was prepared at the apiary of Yangzhou University and provided in a sterile petri dish. On the 2nd day, larvae were transferred to a 48-well plate. One larva and 20 μL feed were placed in a new 48-well and the quantity of food administered was increased each day (20 μL, 30 μL, 45 μL, and 55 μL on days 3, 4, 5, and 6, respectively) (Wu et al., 2017). Once daily for four consecutive days, second instar larvae in the 48-well plate received the artificial food adulterated with 5 ng mg^−1^ carbendazim dissolved in 0.1% DMSO. Larvae in the non-treated group were only fed artificial food containing 0.1% DMSO. On day 7, uric acid crystal deposition in the 48-well plate was observed and the larvae were then transferred to 24-well plates fitted with sterile filter paper. Two days later, the larvae were transferred to new 24-well plates with fresh sterile filter paper. All pupae and larvae were maintained in an incubator at constant 33°C and 70% relative humidity. The brains of five newly emerged workers were dissected off and pooled, frozen in liquid nitrogen, and prepared for RNA extraction and RNA-Seq.

### RNA Isolation, Library Preparation, and Sequencing

Frozen worker honeybee head samples were homogenized in TRIzol reagent (Invitrogen, Carlsbad, CA, United States), and RNA samples were pooled for cDNA library construction. An Agilent 2100 Bioanalyzer (Agilent Technologies, Santa Clara, CA, United States), and a Qubit fluorometer (Invitrogen, United States), were used to determine RNA quantity and quality, respectively. First-strand cDNA was synthesized from mRNA after enrichment by fragmentation with buffer and oligo (dT) magnetic beads (Invitrogen, United States). In brief, random hexamer primers were used for library preparation and amplification. A QIAquick PCR extraction kit (Qiagen, Hilden, Germany), was used to purify the double-stranded cDNA, which was then eluted with EB buffer for end-repair and addition of poly (A). The Illumina HiSeq^TM^ 2000 (Illumina, San Diego, CA, United States), was used to sequence the cDNA library and generate 125, and 150-bp paired-end reads.

### Raw Data Acquisition and Statistical Analysis

A next-generation sequencing quality control (NGS QC) toolkit was used to process raw reads (Patel and Jain, 2012), removing low-quality reads and poly-N. Using Bowtie2 (Langmead and Salzberg, 2012), the resulting clean reads were mapped to the reference *A. mellifera* genome^1^ (Langmead and Salzberg, 2012). Despite sequencing discrepancy and gene length, gene expression data were normalized to reads per kilobase of transcriptome per million mapped reads (RPKM) in order to evaluate the gene expression levels (Mortazavi et al., 2008). An absolute value of log_2_Ratio ≥ 1 (twofold change) and false discovery ratio (FDR) ≤ 0.05 were set as significance thresholds and used to filter the differentially expressed genes (DEGs) (Anders and Huber, 2012).

### Functional Analyses of DEGs

A hierarchical cluster analysis was conducted on the DEGs to identify transcript expression patterns. The hypergeometric distribution model was utilized to conduct Kyoto Encyclopedia of Genes and Genomes (KEGG) pathway and Gene Ontology (GO) enrichment analyses (Anders and Huber, 2012; Roberts and Pachter, 2013).

### Validation by Quantitative Real-Time PCR (qRT-PCR)

Nine candidate differentially regulated carbendazim-sensitive genes with different biological functions were analyzed by qRT-PCR with three biological replicates. The gene-specific primers (GSPs) used are listed in **Supplementary Table S2**. The RNA samples for qRT-PCR validation and for sequencing were randomly and simultaneously collected. Reactions were performed with an ABI 7500 system (Applied Biosystems, Foster City, CA, United States) and SYBR Green (Vazyme, Jiangsu, China). The housekeeping gene β-actin (AB023025) was used as the internal control (Wang et al., 2018). Transcript levels were determined in relation to that of β-actin using the 2^−ΔΔCt^ method (Langmead and Salzberg, 2012), and evaluated for significant differences between treated and non-treated groups using an independent-samples *t*-test in SPSS v. 16.0 (IBM Corp., Armonk, NY, United States) considering *P* < 0.05 as the threshold.

### Juvenile Hormone (JH) and Ecdysone (Ecd) Titers in 6-Day-Old (6 days) Honeybee Larvae

Using the same artificial larval-rearing method, 15 of 6-days honeybee larvae were randomly sampled from control and carbendazim-treated groups for JH and Ecd titer measurements, respectively. Three honeybee colonies were used as biological replicates. For a better understanding of the effect of carbendazim on JH and Ecd of honeybee larvae, the high sublethal concentration of 50 ng mg^−1^ was also adopted.

The test samples were triturated with liquid nitrogen and 5.0 mL methyl alcohol and 3 mL trimethylpentane were added to 1.0 g of each powdered sample. This mixture was vortexed for 5 min and then centrifuged at 10,000 × *g* for 15 min at 4°C. The supernatant was collected, mixed with another 3 mL trimethylpentane, and centrifuged as above. The supernatant was evaporated to dryness with a gentle nitrogen flow at 40°C. The residue was reconstituted with 1.0 mL of acetonitrile/water (2:8, v/v), and this redissolved solution was filtrated through 0.22 μm micropore cellulose membrane and analyzed by ultra high-performance liquid chromatography coupled to quadrupole time-of-flight (UHPLC-Q-TOF/MS) (Sun et al., 2016). The ACQUITY UPLC system (Waters Co., Milford, MA, United States), coupled with the hybrid Q-TOF-mass spectrophotometer SYNAPT HDMS (Waters, St. Michael, United Kingdom), was used, and separation was achieved through an ACQUITY BEH RP18 column (100 mm × 2.1 mm i.d., 3.5 μm particle size) (Waters Co., United States), at the operating flow rate of 0.3 mL min^−1^. Mobile phase A: aqueous solution containing 0.1% formic acid; Mobile phase B: acetonitrile containing 0.1% formic acid. The system was operated in positive electrospray ionization mode (ESI+) with the following typical parameters for mass spectrometry: capillary voltage, 3.0 kV; desolvation temperature 350°C; atomizing pressure, 35 psi; nitrogen flow, 9 L min^−1^; scanned range, *m/z* = 200–2000. The independent-sample *t*-test in SPSS v. 16.0 (IBM Corp., Armonk, NY, United States) was adopted for evaluating significant differences between treated and non-treated groups, considering a threshold of *P* < 0.05.

## Results

### Raw RNA-Seq Data Analysis

Transcriptome sequencing statistics for all samples are summarized in **Supplementary Table S3**. Approximately, 48 million clean, single-end mRNA reads were generated. Of these,> 80% were successfully aligned to the honeybee reference genome. The expression levels of the genes identified and expressed in the heads of the newly emerged workers exposed to carbendazim or non-treated were compared based on RPKM (Trapnell et al., 2010). The reported sequencing data has been approved and assigned to SRA (Sequence Read Archive) database (SRA accession number: SRP158178). Correlation analyses among the three biological replicates of each sample (**Supplementary Table S4**) indicated high sequencing reliability.

### Screening and Functional Analyses of DEGs

Based on *P* < 0.05 and log_2_Ratio ≥ 1, 247 DEGs were identified between the carbendazim-treated and non-treated (control) samples. Of these, 187 were upregulated and 60 were downregulated in the carbendazim-treated group compared to the control group (**Supplementary Table S5**).

**FIGURE 1:**
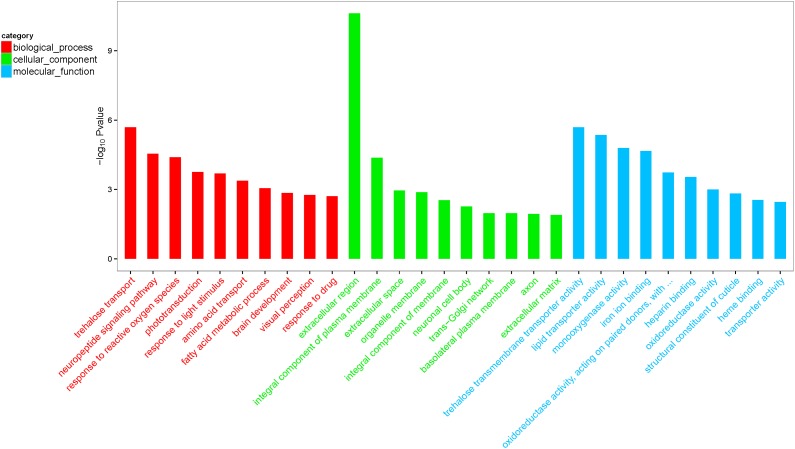
Gene ontology (GO) top 30 terms enriched by differentially expressed genes (DEGs). The results are summarized in three main categories: biological process, cellular component, and molecular function. The *x*-axis indicates the second term of GO and the *y*-axis indicates gene percentage.

**FIGURE 2:**
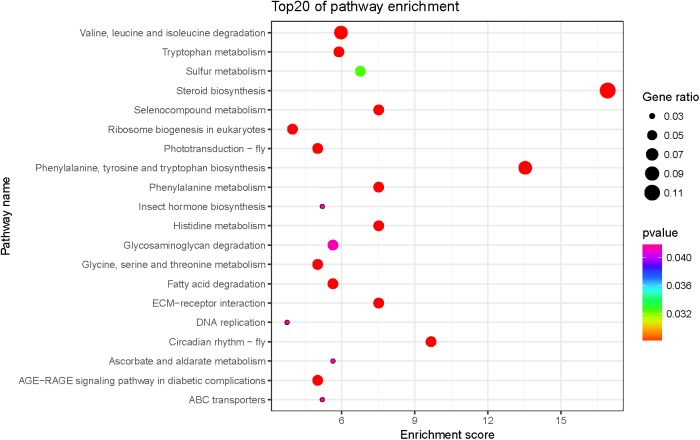
Top 20 significantly enriched Kyoto Encyclopedia of Genes and Genomes (KEGG) pathways of DEGs. Color and gene ratio indicate the *P*-value and the ratio of genes within each pathway, respectively. The *x*-axis indicates the enrichment score of KEGG and the *y*-axis indicates the name of the top 20 pathways.

The biological functions of the 247 DEGs were classified according to the GO database, and were organized into three main categories: molecular function, biological process, and cellular component. The 692 significantly enriched GO terms successfully mapped (*Q* < 0.05), and the top 10 enriched terms per category were selected to further elucidate the functions of the DEGs. These included the activities of lipid transporter, monooxygenase, and oxidoreductase, as well as cuticle composition and heme-binding in the molecular function category, and neuropeptide signaling pathway, response to reactive oxygen species, fatty acid metabolism, brain development, visual perception, and drug response in the biological process category (**Figure 1**). The biochemical pathways of the DEGs were investigated using the KEGG database. The top 20 enriched KEGG pathways included fatty acid valine, leucine, and isoleucine degradation, tryptophan, glycine, serine, and threonine metabolism, and eukaryotic ribosome biogenesis (**Figure 2**).

### Validation of Target DEGs by qRT-PCR

**FIGURE 3:**
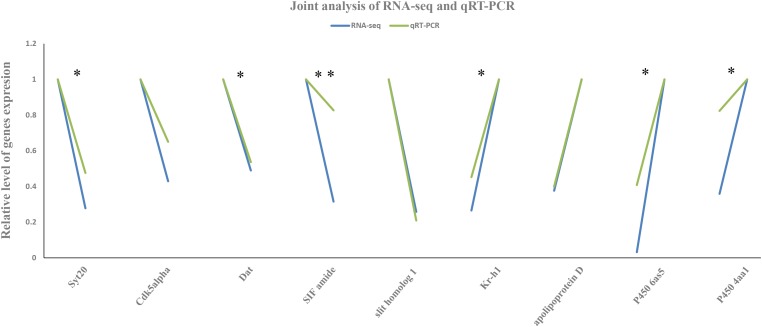
Joint analysis of RNA sequencing (RNA-Seq) and quantitative real-time PCR (qRT-PCR). The *x*-axis represents the functional gene name and the *y*-axis the relative level of their expression by RNA-Seq and qRT-PCR. Green and blue represent the values obtained from RNA-seq and qRT-PCR methods, respectively. All genes with significantly different expression between the carbendazim-treated and control groups are indicated using asterisks: ^∗^*P* < 0.05, ^∗∗^*P* < 0.01 (independent-samples *t*-test).

The expression patterns of the nine DEGs selected for validation by qRT-PCR were consistent with that from RNA-Seq (**Figure 3** and **Supplementary Table S2**). Relative expression levels of these DEGs provided a better comparison and the highest expression levels were set to 1 (Wang et al., 2018). Their agreement indicated that the abundance of the Illumina reads closely mirrored the actual expression levels of the DEGs.

### Titer of JH and Ecd in 6 Days Larvae After Carbendazim Exposure

**FIGURE 4:**
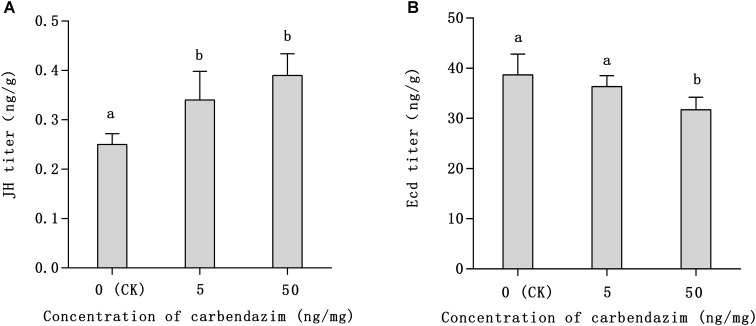
Titers of juvenile hormone (JH; **A**) and ecdysone (Ecd; **B**) in 6-day-old larvae after carbendazim exposure and in the control group. Data presented in the table are mean ± standard error. The *x*-axis represents the control group [larvae fed artificial food containing 0.1% dimethyl sulfoxide (DMSO)] and the treated groups (larvae fed with artificial food adulterated with 5 or 50 ng mg^−1^ of carbendazim dissolved in 0.1% DMSO). Different letters above bars represent significant differences (independent-samples *t*-test, *P* < 0.05).

Notably, larvae in the carbendazim-treated group needed more time to shift into the dormant pupal state than larvae in the non-treated group, which was considered abnormal we observed. The JH and Ecd titers of 6 days honeybee larvae are shown in **Figure 4**. As depicted, the JH titers of 6 days larvae treated with carbendazim increased by 1.36 and 1.56 times in relation to that of control larvae (**Figure 4A**), respectively (*P* < 0.05). The Ecd titers showed an opposite pattern (**Figure 4B**), as that of the treated group were lower than that of the control group (82%), especially in honeybee larvae exposed to the highest carbendazim stress concentration (50 ng mg^−1^; *P* < 0.05). These results indicated that the tested concentrations of carbendazim can promote JH synthesis and inhibit the secretion of Ecd in 6 days larvae.

## Discussion

Rearing honeybee larvae *in vitro* is of great importance for research on pathogens and risk assessment (Crailsheim et al., 2013). In the present study, honeybees were exposed to the fungicide carbendazim to assess and evaluate its sublethal effects. The examined honeybees were raised *in vitro* from the larval stage in order to eliminate the effect of the interfering factors associated with hive rearing. Suitable carbendazim post-exposure risk evaluation samples were then prepared for RNA-Seq and qRT-PCR. Our previous *in vitro* larval-rearing revealed a 75 emergence rate, which was acceptable (Brodschneider et al., 2009; Aupinel et al., 2010; Crailsheim et al., 2013; Wu et al., 2017), and demonstrated the reliability of the present results.

Among the 247 DEGs identified after carbendazim exposure (5 ng mg^−1^), using a rigorous quantitative strategy via RNA-Seq, 187 were upregulated and 60 were downregulated in the treated group compared with the control group. The DEGs found in the present study reflect the multifaceted influence of carbendazim on honeybee physiology. Interestingly, results showed that carbendazim exposure at the larval stage significantly changed the expression of genes encoding major royal jelly proteins (MRJPs) in adult honeybee brain. In fact, MRJP1, MRJP3, MRJP4, MRJP5, and MRJP6 were upregulated in the carbendazim-exposed group (**Supplementary Figure S1**). A previous study showing the expression of MRJP1–9 in honeybee brains suggested that these genes might have other functions in addition to regulating royal jelly biosynthesis (Buttstedt et al., 2013). For example, MRJP1 has antimicrobial properties (Fontana et al., 2004; Rosmilah et al., 2008). To the best of our knowledge, the present study is the first to show that carbendazim exposure in honeybee upregulates MRJPs expression, a finding that merits further investigation.

In 187 upregulated genes library, 59 of these genes (approximately 31.5%), were grouped into receptor and transporter categories. Among these 59 genes, 43 were upregulated and 16 were downregulated (**Supplementary Figure S1**). The upregulation of these genes in response to carbendazim exposure might induce toxicant metabolism (Wu et al., 2017). For example, carbendazim stress upregulated the expression of cytochrome P450s, such as P450 6AS5 (GB49890) and 4aa1 (GB49626). The members of the CYP450 family 4 are responsible for the metabolism of endogenous substrates, including pheromones and hormones, whereas members of the CYP450 family 6 are involved in xenobiotic detoxification and transport (Corona and Robinson, 2006). On the other hand, one of the 16 transporter and receptor genes downregulated by carbendazim exposure encoded apolipoprotein D (GB54020). Therefore, this fungicide may adversely affect honeybee receptor-associated lipid metabolism. Other functional genes like G-protein coupled receptor (GB48344), which have been demonstrated to enhance spatial recognition memory in rats, was also downregulated, and this suppression might cause orientation and identification disorders in honeybee after carbendazim exposure (Riedel et al., 2003; Hawley et al., 2014). Because, both receptor and transporter proteins are essential for metabolism and development the changes in the expression of the genes encoding them might cause adverse effects, ultimately destroying the physiological system.

Transcriptome analyses indicated that sublethal carbendazim exposures in honeybees either upregulated or downregulated genes involved in immunity, detoxification, sensory processing, brain development, and metabolism. The variability of gene expression in response to carbendazim exposure suggests that this fungicide might influence multiple aspects of honeybee physiology, threatening their survival. For instance, in carbendazim-exposed honeybees the number of transcripts of Cyp4aa1-like, Cyp6as5, abaecin (GB18323), and defensin 1 (GB41428), which are involved in detoxification and immunity, was 31-, 2.8-,13-, and 197.7-fold that in control honeybees, respectively, suggesting that defense was initiated upon fungicide exposure. Importantly, certain genes associated with brain development were significantly downregulated after carbendazim exposure. These included synaptotagmin 20 (Syt20, GB54319), cyclin-dependent kinase 5 activator 1 (Cdk5alpha, GB55798), dopamine transporter (DAT, GB40867), SIF amide (GB40093), and slit homolog 1 (GB43897) (**Supplementary Figure S1**).

Synaptotagmins are synaptic proteins, which play important roles in calcium-mediated, depolarization-induced exocytosis, neurotransmitter release, and hormone secretion. These processes are essential for the transmission of neural signals and brain development (Fernandez-Chacon et al., 2001; Chang et al., 2018). Because synaptotagmins share a similar domain structure and a high degree of homology (Huang et al., 2017; Saheki, 2017), Syt20 might harbor similar functionality although its characteristics have not been studied to date. The specific activator of cyclin-dependent kinase 5 (CDK5), Cdk5alpha, is an important molecule for nervous system development and function and for neurodegenerative disorder pathogenesis (Kobayashi et al., 2014; Papadopoulou et al., 2015). Mutations in *Cdk5* have been associated with neuronal injury and neurodegeneration and lead to the failure of synaptic cognition and modeling (Lai et al., 2015; Yousuf et al., 2016). Dopamine is an important neurotransmitter in both invertebrate and vertebrate nervous systems. It is widely distributed in the honeybee brain and it is associated with behavior regulation and olfactory memory (Schürmann et al., 1989; Beggs and Mercer, 2009; Mustard et al., 2010). Dopamine transporter removes dopamine from the synaptic cleft and deposits it into the surrounding cells. Therefore, DAT terminates the neurotransmitter signal and helps elucidate the primary mechanism of dopamine. Previous studies have shown that the queen regulates worker behavior and physiology via certain molecular mechanisms, especially dopamine signal pathways (Beggs et al., 2007; Beggs and Mercer, 2009). Therefore, DAT downregulation may affect honeybee brain development and can induce rebellious behavior in workers and colony disorder. Evidence indicates that, under axonal guidance, slit homolog 1 participates in nervous system development, especially cortex development (Hao et al., 2001; Farmer et al., 2008). The highly conserved neuropeptide SIF amide plays an important role in the olfactory neural pathway and has a neuromodulatory role in combining tactile, olfactory, and visual inputs (Verleyen et al., 2004, 2009). In the present study, the expression of the genes encoding these proteins related to brain development was validated by qRT-PCR (**Figure 3** and **Supplementary Table S2**). The significant agreement of RNA-Seq and qRT-PCR results indicated that Illumina sequencing results closely mirrored the actual expression level of these genes and emphasized the negative effect of carbendazim on honeybee brain. In the present study, GO enrichment analysis showed that the expression of the genes involved in brain development and on neuropeptide signaling pathway was significantly decreased in honeybees exposed to carbendazim (GO:0007420 and GO:0007218). These fluctuations in gene expression may impair honeybee brain function and possibly lead to the collapse of the whole colony.

It is widely accepted that exogenous adverse factors can delay insect development, as found in honeybee (Czoppelt and Rembold, 1988; Wu et al., 2014). In the present study, we found that carbendazim exposure delays insect development. Larvae in carbendazim-treated groups needed more time to shift into the dormant pupal state and this abnormality was more obvious with increasing carbendazim concentrations (50 ng mg^−1^). The degree of honeybee development is often regulated by hormones, mainly JH and Ecd (Corona et al., 2007; Schulz et al., 2014). The titers of JH and Ecd in 6 days larvae found here (**Figure 3**) were higher and lower, respectively, in the carbendazim-treated group than in the control group, thus confirming that this fungicide accounted for the delay in development. The Kruppel-like homolog 1 (*Kr-h1*), a hormone regulatory gene, was one of the DEGs found in the present study. It is a key regulator of insect molting and metamorphosis and a major effector in JH signaling (Pecasse et al., 2000; Minakuchi et al., 2008). During insect development, Kr-h1 functions as a transcriptional repressor on the neurogenesis of the mushroom body and photoreceptor maturation, which is considered an interaction with the JH signaling (Shi et al., 2007; Fichelson et al., 2012; Kayukawa et al., 2012). In *Drosophila melanogaster*, Kr-h1 delays larval development and alters lipid metabolism (Ping et al., 2017). Furthermore, previous research reported that structural cuticle genes were downregulated as the insect entered the diapause maintenance phase of diapause development (Yocum et al., 2009). In the present study, members of a cuticle protein family: apidermin 1 (GB53115), apidermin 2 (GB53119), apidermin 3 (GB53114), cuticular protein 5 (GB40299), and cuticular protein 19 (GB50453) tended to be downregulated in the brains of newly emerged adult honeybees after carbendazim exposure (**Supplementary Figure S1**). This downregulation of cuticular genes might be a morphological manifestation of the delay in development. Thus, carbendazim-induced Kr-h1 upregulation may cause JH disorder and delay honeybee development.

Some other functional genes were found to change their expression patterns after carbendazim exposure. Kin of IRRE-like protein 2 (Kirrel2, GB45525) encodes pancreatic islet β-cell-expressed protein. It is an integral part of the extracellular adhesion scaffold required for normal basal insulin secretion (Yesildag et al., 2015). Insect insulin-like peptides and their signal pathways play important roles in metabolism, longevity, growth, and development (Antonova et al., 2012; Fridell, 2013; Yesildag et al., 2015). Queen brain-selective protein-1 (QBP-1, GB45741) plays a role in determining honeybee longevity (Grönke and Partridge, 2010), and its isoforms function in phase transition (Vanhems et al., 1990; Claeys et al., 2005) in locusts. Therefore, downregulation of these genes might reflect the impairment of certain unmanifested functions.

Overall, the results obtained here demonstrate that sublethal doses of carbendazim hinder growth and development and may destabilize and impede the development of honeybee colonies. Thus, they should be used to inform beekeepers and farmers about the effects of carbendazim on honeybees and provide guidance in using this fungicide carbendazim in agriculture reasonably.

## Ethics Statement

This study was carried out in accordance with the recommendations of ‘Guidelines for laboratory animals of Yangzhou university, Yangzhou university animal welfare committee’ with written informed consent from all subjects. All subjects gave written informed consent in accordance with the Declaration of Helsinki. The protocol was approved by the ‘Yangzhou university animal welfare committe.’

## Author Contributions

KW designed the study and carried out the data analysis. R-LF and W-NJ participated in drafting the manuscript. W-WZ, X-MC, and SW participated in sample collection. LY and TJ provided advice on data analyses and helped to drafting the manuscript. F-CG and G-HC were responsible for answering questions and language Edit in the revision process.

## Conflict of Interest Statement

The authors declare that the research was conducted in the absence of any commercial or financial relationships that could be construed as a potential conflict of interest.

## Footnotes

1 ftp://ftp.ncbi.nlm.nih.gov/genomes/all/GCF/000/002/195/GCF_000002195.4_Amel_4.5/GCF_000002195.4_Amel_4.5_genomic.fna.gz

## Supplementary Material

FIGURE S1 Correlation coefficient of samples. A heat map representation of the correlation matrix of sequencing samples.

TABLE S1 Survival Rates. Survival rates of control and 5 ng mg^−1^ carbendazim-treated groups (Chi-Square (χ^2^ Tests).

TABLE S2 Gene-specific primers. Specific primers of functional gene used for qRT-PCR.

TABLE S3 Summary of mapping result of RNA-seq. All genome mapping statistics.

TABLE S4 All differentially expressed genes. An absolute value of log_2_Ratio ≥ 1 (two-fold change) and false discovery ratio (FDR) ≤ 0.05 were set as significance thresholds and used to filter the differentially expressed genes (DEGs).

TABLE S5 Functional gene in DEGs. Different kinds of functional gene among DEGs, including major royal jelly proteins, transporter and receptor, cuticle structure and Brain development related genes.
