# Supplementary figures and images for: Transcriptome Analysis of Newly Emerged Honeybees Exposure to Sublethal Carbendazim During Larval Stage
Source: Front Genet. 2018 Oct 8;9:426. doi: 10.3389/fgene.2018.00426 (PMC6186791; doi:10.3389/fgene.2018.00426)

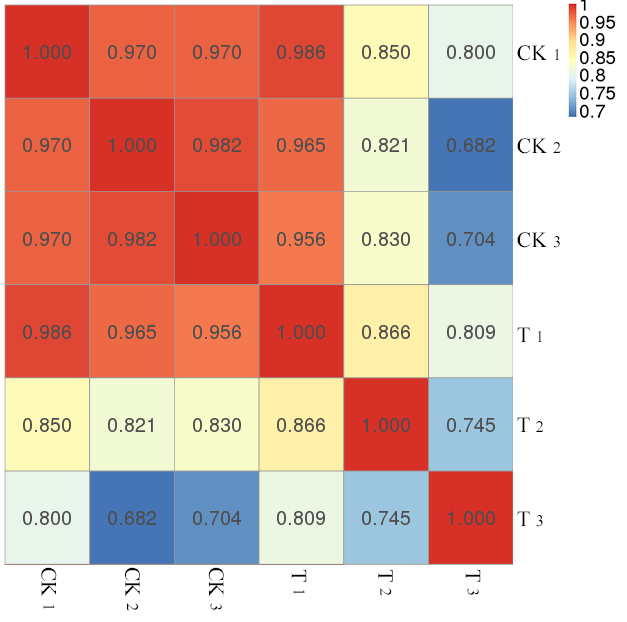

Supplement: FIGURE S1 — Correlation coefficient of samples. A heat map representation of the correlation matrix of sequencing samples. [file Image_1.TIF]
